# Ferric citrate is a safe and digestible source of iron in broilers and piglets

**DOI:** 10.7717/peerj.12636

**Published:** 2021-12-22

**Authors:** Klaus Männer, Hannah Lester, Eliana Henriquez-Rodriguez

**Affiliations:** 1 Fachbereich Veterinärmedizin, Freie Universität Berlin, Institut für Tierernährung, Berlin, Germany; 2 Regulatory Affairs, Pen & Tec Consulting S.L.U., Sant Cugat del Vallés, Barcelona, Spain

**Keywords:** Safety, Digestibility, Poultry, Weaned piglets, Mineral, Organic, Haematology

## Abstract

**Background:**

Iron (Fe) is traditionally supplemented in poultry and swine diets using inorganic forms (*e.g*. sulfates, oxides). However, research suggests that organic sources are more beneficial due to greater bioavailability. In this paper, we present results from four studies aimed at assessing ferric citrate (CI-FER™, Akeso Biomedical Inc., Burlington, MA, USA) as a safe and effective source of Fe for broilers and piglets.

**Methods:**

A total of four studies were performed in Germany following standard farming practices for each species. One study in day-old broiler chicks and one study in weaned piglets were designed as target animal safety studies where animals were randomly allocated to one of three treatment groups: a negative control group, the proposed dose group and a multifold dose group. Broilers and pigs were fed the experimental diets for 35 and 42 days, respectively. In each study, average daily feed intake, average daily weight gain and feed conversion ratio were measured, and blood samples were taken at study end for routine biochemistry and haematology. The other two studies were designed to evaluate different sources of dietary Fe for weaned piglets bred and managed under standard farm conditions. All piglets received routine Fe injections (200 mg Fe dextran, intramuscular) on day 3 of age, as well as the experimental diets for 42 days. In both studies, performance parameters were measured. In one study, Fe digestibility and serum Fe, superoxide dismutase and haptoglobin were also measured. In all studies, the general health status of the animals was monitored daily and all culls and mortality recorded. Each study followed a complete randomised block design.

**Results:**

In broilers, ferric citrate was well tolerated up to 2,000 mg/kg feed (×10 the recommended inclusion rate) and no adverse effects on growth, blood parameters or mortality were observed. In piglets, ferric citrate was well tolerated up to 5,000 mg/kg feed (×10 the recommended inclusion rate) with no adverse effects on growth, blood parameters or mortality. In addition, piglets fed ferric citrate performed significantly better than animals fed the negative control diet (containing only endogenous Fe) and those fed inorganic forms of Fe. Moreover, piglets fed ferric citrate demonstrated improved Fe digestibility and improved oxidative status. Altogether, these findings show that ferric citrate is a safe and easily digestible source of dietary Fe for broilers and piglets.

## Introduction

Intensive farming production systems require farmers to pay careful attention to the main factors that determine profitability, such as genetics, health, management and nutrition. In relation to the latter, iron (Fe) as a trace mineral is particularly important since it is involved in oxygen and electron transport, deoxyribonucleic acid (DNA) synthesis, energy metabolism and gene regulation, among others (Beard, 2001). In poultry, the fast-growing modern broiler is sensitive to dietary deficiencies of Fe which can cause severe anaemia with reduction of packed cell volume (PCV), haemoglobin and body weight (Tako, Rutzke & Glahn, 2010; Marks & Kendall, 2020). In suckling piglets, it is well established that insufficient Fe intake results in Fe deficiency, anaemia and increased early morbidity and mortality (Kernkamp, Clawson & Ferneyhough, 1962). Young pigs are commonly at risk of Fe deficiency as the piglet is born with a limited Fe store of approximately 50 mg of Fe, most of which is incorporated in haemoglobin (Venn, McCance & Widdowson, 1947). Sow milk is a poor source of Fe and only provides sucking piglets with approximately 1 mg of Fe per day (Venn, McCance & Widdowson, 1947). Moreover, the modern pig is selected for rapid growth and piglets double their weight in the first seven days of life, requiring increased Fe as a larger volume of blood is needed to support and maintain tissue growth (Peters & Mahan, 2008). The daily Fe requirement for piglets is ca. 7 mg and so the limited store of Fe at birth, coupled with the limited Fe obtained from sow milk, means that, without exogenous Fe supplementation, fast-growing piglets become Fe deficient within the first week of life (Venn, McCance & Widdowson, 1947; Murphy, Friendship & Dewey, 1997; Starzynski et al., 2013). Intramuscular injection with Fe dextran (200 mg) in the first three days is used routinely to prevent Fe deficiency in piglets (Svoboda & Drabek, 2005). However, the introduction of new genetic lines and management practices have raised questions about the efficacy and safety of this method with some authors reporting that additional Fe supplementation is required depending on Fe requirements and the availability of Fe sources (Svoboda, Vanhara & Berlinska, 2017).

Traditionally, trace minerals such as Fe are supplemented using inorganic forms (*e.g*. sulfates, oxides) but research suggests that the use of organic sources is beneficial due to their greater bioavailability, given that organic compounds can reduce interference from agents that form insoluble complexes with ionic trace elements (Abdallah, El-Husseiny & Abdel-Latif, 2009; Kumar-Mohanta & Kumar-Garg, 2014). This hypothesis is supported by studies such as those of Shinde et al. (2011) and Arnaudova-Matey et al. (2013), who showed that, compared with inorganic forms, broiler diets containing organic sources of Fe increase Fe concentration in different tissues and reduce Fe excretion. Earlier studies in pigs also demonstrated organic forms of Fe to be safe and efficacious (Furugouri & Kawabata, 1975).

In this paper, we present the results from four experiments aimed at assessing the effects of ferric citrate (CI-FER™; Akeso Biomedical Inc., Burlington, MA, USA), an organic iron chelate, as a safe and effective source of Fe for broilers and weaned piglets bred and managed under standard farm conditions.

## Materials & Methods

### Study location & quality standards

The four studies were performed at the Institute of Animal Nutrition, Department of Veterinary Medicine, Freie University of Berlin (Berlin, Germany). All studies were conducted according to the Animal Welfare Act of Germany approved by the local state office of occupational health and technical safety (Landesamt für Gesundheit und Soziales, LaGeSo, no. A 0439/17).

### Experimental design and diets

The test item ferric citrate contains ca. 17% Fe, hence at 200, 2,000, 500 and 5,000 mg/kg delivers 34, 340, 85 and 850 mg Fe/kg feed, respectively.

#### Study A

The objective of this study was to assess the tolerance and efficacy of ferric citrate as a source of dietary Fe for broilers bred and managed under standard farm conditions. A total of 480 pre-sexed, one-day-old male broilers (Cobb 500) were allocated to three treatment groups (8 replicate pens/treatment with 20 birds/pen) in a complete randomised block design: Control (T1), T1 + ferric citrate at 200 mg/kg feed (T2), and T1 + ferric citrate at 2,000 mg/kg feed (T3). During the 35-day trial period, animals were housed in the pens of a poultry barn and had *ad libitum* access to water and mash feeds - starter diet from 0–14 days and grower diet from 15–35 days. The pens measured 2.2 × 2.0 m, providing 0.22 m^2^ per bird, with bedding of softwood shavings. The basal diets, other than Fe supplements, were calculated to be iso-nutritive, meeting or slightly exceeding nutritional requirements for broilers as recommended by the Society of Nutritional Physiology (1999). Fe was not supplemented in the T1 Control diet. The basal diets (T1 Control) analysed at 77.8 and 80.4 mg endogenous Fe/kg feed for starter and grower diets respectively. The ferric citrate contained microtracers (MicroTracers, San Francisco, CA USA, at inclusion rate of 10%), used as a marker. Microtraced ferric citrate was added to the basal diets to replace the Tixosil^®^ (>97% colloidal silica) in the control diet. Feed samples were analysed for dry matter, crude protein, crude fat, ash and Fe using the Association of German Agricultural Analytic and Research Institutes methods (VDLUFA (1993) methods), broadly similar to the Association of Official Analytical Chemists methods (AOAC, 2000). Fe analysis (in this and the piglet studies) was performed following the protocol for atomic absorption spectroscopy (Contr AA 700; Analytik Jena, Jena, Germany). Details of diet composition, analysed nutritional values and microtracer recovery are presented in Tables 1 and 2, respectively. All animals were vaccinated against Newcastle disease and infectious bronchitis at the hatchery.

**Table 1:** Study A: composition and calculated analysis of starter (0–14 d of age) and grower diets (15–35 d of age).

Ingredient	Units	Starter diet			Grower diet		
		T1 Control	T2 Ferric citrate 200 mg/kg	T3 Ferric citrate 2,000 mg/kg	T1 Control	T2 Ferric citrate 200 mg/kg	T3 Ferric citrate 2,000 mg/kg
Maize	%	31.77	31.77	31.77	30.97	30.97	30.97
Soybean meal (CP 47%)		33.31	33.31	33.31	29.57	29.57	29.57
Wheat		25.20	25.20	25.20	29.30	29.30	29.30
Soybean oil		5.20	5.20	5.20	5.80	5.80	5.80
Monocalcium-phosphate		1.50	1.50	1.50	1.38	1.38	1.38
Premix^1^		1.20	1.20	1.20	1.20	1.20	1.20
Limestone		1.03	1.03	1.03	1.03	1.03	1.03
DL-Methionine		0.31	0.31	0.31	0.23	0.23	0.23
L-Lysine HCL		0.19	0.19	0.19	0.23	0.23	0.23
L-Threonine		0.09	0.09	0.09	0.09	0.09	0.09
Tixosil^®^ colloidal silica		0.20	0.18	0	0.20	0.18	0
Ferric citrate		0	0.02	0.20	0	0.02	0.20
TOTAL		100	100	100	100	100	100
AME_N_^2^	MJ/kg	12.42	12.42	12.42	12.73	12.73	12.73
AME_N_^3^		12.65	12.65	12.65	12.90	12.90	12.90
Crude protein	g/kg	215.60	215.60	215.60	200.90	200.90	200.90
Lysine		12.70	12.70	12.70	12.00	12.00	12.00
Methionine		6.20	6.20	6.20	5.20	5.20	5.20
Methionine/Cysteine		9.70	9.70	9.70	8.50	8.50	8.50
Tryptophan		2.60	2.60	2.60	2.40	2.40	2.40
Threonine		8.70	8.70	8.70	8.10	8.10	8.10
Valine		1.00	1.00	1.00	9.30	9.30	9.30
Arginine		1.41	1.41	1.41	12.90	12.90	12.90
Crude fibre		26.30	26.30	26.30	25.70	25.70	25.70
Crude fat		73.80	73.80	73.80	79.80	79.80	79.80
Starch		359.10	359.10	359.10	380.70	380.70	380.70
Sugars		42.60	42.60	42.60	39.90	39.90	39.90
Calcium		9.00	9.00	9.00	8.80	8.80	8.80
Total phosphorus		7.20	7.20	7.20	6.70	6.70	6.70
Sodium		1.70	1.70	1.70	1.70	1.70	1.70

**Notes:**

1 Per kg: 600,000 I.U. Vit. A (acetate); 120,000 I.U. Vit. D_3_; 6,000 mg Vit. E (α-tocopherol acetate); 200 mg Vit. K_3_ (MSB); 250 mg Vit. B_1_ (mononitrate); 420 mg Vit. B_2_ (cryst. riboflavin); 300 mg Vit. B_6_ (pyridoxin-HCl); 1,500 μg Vit. B_12_; 3,000 mg niacin (niacinamide); 12,500 μg biotin (commercial, feed grade); 100 mg folic acid (cryst., commercial, feed grade); 1,000 mg pantothenic acid (Ca d-pantothenate); 60,000 mg choline (chloride); 5,000 mg zinc (zinc sulphate); 6,000 mg manganese (manganous oxide); 1,000 mg copper (copper oxide); 45 mg iodine (calcium-iodate); 20 mg selenium (sodium-selenite); 140 g sodium (NaCl); 55 g magnesium (magnesium sulphate); carrier: calcium carbonate (calcium min 38%); no added Fe.

2 Estimated according to equation of WPSA (1984) (by using crude nutrients).

3 According to INRA (2004).

**Table 2:** Study A: key analysed values of starter and grower feeds (as is).

Treatment	Dry matter (%)	Crude protein (%)	Crude fat (%)	Ash (%)	Fe (mg/kg)	Microtracer (% recovery)	Calculated ferric citrate content (mg/kg feed)^1^
Starter diet (0–14 d)							
T1 Control	90.56	21.83	7.42	5.81	77.8	NA	–
T2 Ferric citrate, 200 mg/kg feed	90.71	21.85	7.39	5.78	116.5	92 %	184
T3 Ferric citrate, 2,000 mg/kg feed	90.65	21.79	7.44	5.80	409.3	71%	1,420
Grower diet (15–35 d)							
T1 Control	90.79	20.28	8.42	5.34	80.4	NA	–
T2 Ferric citrate, 200 mg/kg feed	90.77	20.25	8.35	5.30	124.5	84%	168
T3 Ferric citrate, 2,000 mg/kg feed	90.80	20.30	8.39	5.33	428.2	78%	1,560

**Notes:**

NA = Not applicable.

1 Calculated ferric citrate = % recovery of microtracer x ferric citrate dose.

#### Study B

The objective of this study was to evaluate the safety of ferric citrate as a source of dietary Fe for weaned piglets bred and managed under standard farm conditions. A total of 144 healthy weaned piglets (25 days of age) from a single weaning batch of ca. 310 piglets (Danbred x Piètrain) were used. Piglets received routine Fe injections (200 mg Fe dextran, intramuscular) on day 3 of age (as per standard farming practice) and creep feed (without addition of Fe) from day 8 to 24 of age, respectively. Details of the diet composition are presented in Table 3. The analysed Fe content in the creep feed was 59.4 mg/kg (Table 4). The piglets were allocated to 3 treatment groups (T1 - negative control; T2 - 500 mg ferric citrate/kg feed; T3 - 5,000 mg ferric citrate/kg feed) balanced as far as possible for body weight (BW), litter origin, and gender in 24 pens (12 male and 12 female pens, 6 piglets per pen, 8 pens per treatment) from 25 to 66 d of age (42-d feeding period). Starter mash was fed for 14 days, and grower mash was fed subsequently, until study end (42 days post-weaning). The basal diets (T1 Control) contained 69.2 and 76.2 mg endogenous Fe/kg feed for starter and grower diets, respectively, and were Fe deficient compared to the recommended 100 mg Fe/kg for growing piglets fed an *ad libitum* diet (based on 90% dry matter) between 5–25 kg of weight (National Research Council (NRC), 2012, Table 4). The ferric citrate used contained microtracer (MicroTracers, San Francisco, CA USA, at an inclusion rate of 10%) as a marker. Microtraced ferric citrate was added to the basal diets instead of Tixosil^®^ (>97% colloidal silica). Feed samples were analysed for dry matter, crude protein, crude fat, ash and Fe using VDLUFA (1993) methods. Details of analysed nutritional values and microtracer recovery are shown in Table 4. Post-weaning piglets had *ad libitum* access to feed (mash form) and water (drinking bowls). The pens were equipped with solid partitions and slotted floors in a climate-controlled post-weaning house, with 2 rows of pens separated by a central aisle. Pen measured 3.2 × 2.0 m, providing 1.1 m^2^ per animal.

**Table 3:** Study B: composition and calculated analysis of starter (0–14 d of age) and grower diets (15–42 d of age).

Ingredients	Units	Creep feed	Starter diet			Grower diet		
			T1 Control	T2 Ferric citrate, 500 mg/kg	T3 Ferric citrate 5,000 mg/kg	T1 Control	T2 Ferric citrate, 500 mg/kg	T3 Ferric citrate 5,000 mg/kg
Corn	%	50.57	63.01	63.01	63.01	70.55	70.55	70.55
Soybean meal (CP 49%)		24.38	19.18	19.18	19.18	21.51	21.51	21.51
Skimmed milk powder		20.00	10.00	10.00	10.00	–	–	–
Mineral & vitamin premix^1^		1.20	1.20	1.20	1.20	1.20	1.20	1.20
Calcium carbonate		1.15	1.38	1.38	1.38	1.43	1.43	1.43
Monocalcium phosphate		1.10	1.40	1.40	1.40	1.60	1.60	1.60
Soybean oil		1.00	0.54	0.54	0.54	1.17	1.17	1.17
L-lysine HCL		0.30	0.60	0.60	0.60	0.57	0.57	0.57
DL-methionine		0.20	0.23	0.23	0.23	0.17	0.17	0.17
Cellulose		–	1.00	1.00	1.00	1.00	1.00	1.00
L-tryptophan		0.10	0.10	0.10	0.10	0.08	0.08	0.08
Tixosil^®^ colloidal silica (>97% silicon dioxide)		0	0.550	0.495	0	0.550	0.495	0
Ferric citrate		0	0	0.055	0.500	0	0.055	0.500
TOTAL		100	100	100	100	100	100	100
AME_N_^2^	MJ/kg	13.91	13.42	13.42	13.42	13.40	13.40	13.40
Crude protein	%	24.00	20.00	20.00	20.00	18.00	18.00	18.00
Lysine		1.65	1.51	1.51	1.51	1.29	1.29	1.29
Methionine		–	0.57	0.57	0.57	0.44	0.44	0.44
Methionine/Cysteine		0.98	0.87	0.87	0.87	0.74	0.74	0.74
Tryptophan		0.37	0.30	0.30	0.30	0.25	0.25	0.25
Threonine		0.96	0.94	0.94	0.94	0.82	0.82	0.82
Valine		–	–	–	–	–	–	–
Arginine		–	–	–	–	–	–	–
Crude fibre		1.94	2.74	2.74	2.74	2.96	2.96	2.96
Crude fat		3.65	3.50	3.50	3.50	4.39	4.39	4.39
Starch		–	–	–	–	–	–	–
Crude ash		–	5.63	5.63	5.63	5.28	5.28	5.28
Calcium		0.96	0.95	0.95	0.95	0.88	0.88	0.88
Phosphorus		0.75	0.45	0.45	0.45	0.40	0.40	0.40
Digestible phosphorous		0.48	–	–	–	–	–	–
Sodium		2.20	0.24	0.24	0.24	1.76	1.76	1.76

**Notes:**

1 Per kg premix: 400,000 I.U. vit. A (acetate); 120,000 I.U. vit. D3; 8,000 mg vit. E (a-tocopherol acetate); 200 mg vit. K3 (MSB); 250 mg vit. B1 (mononitrate); 420 mg vit. B2 (cryst. riboflavin); 2,500 mg niacin (niacinamide); 400 mg Vit. B6 (HCl); 2,000 μg vit. B12; 25,000 μg Biotin (commercial, feed grade); 1,000 mg pantothenic acid (Ca d-pantothenate); 100 mg folic acid (cryst. commercial feed grade); 80,000 mg choline (chloride); 5,000 mg Zn (sulphate); 6,000 mg Mn (sulphate);1,000 mg Cu (sulphate-pentahydrate); 20 mg Se (Na-selenite); 45 mg I (Ca-iodate); 130 g Na (NaCl); 55 g Mg (sulphate).

2 Metabolisable Energy, calculated as per DLG (2013). No added Fe to creep feed or to basal post-weaning diets.

**Table 4:** Study B: key analysed values of starter and grower feeds (as is).

Treatment	Dry matter (%)	Crude protein (%)	Crude fat (%)	Ash (%)	Fe (mg/kg)	Microtracer (% recovery)	Calculated ferric citrate content (mg/ kg feed)^1^
Creep feed	91.70	24.84	3.42	6.24	59.4	NA	–
Starter diet (0–14 d)							
T1 Control	91.24	20.10	3.64	5.70	69.2	NA	–
T2 Ferric citrate, 500 mg/kg feed	90.93	20.06	3.60	5.68	162.4	97	485
T3 Ferric citrate, 5,000 mg/kg feed	90.18	20.11	3.62	5.40	1,012	93	4,650
Grower diet (15–42 d)							
T1 Control	90.42	18.62	4.47	5.30	76.2	NA	–
T2 Ferric citrate, 500 mg/kg	90.26	18.62	4.48	5.32	168.7	98	490
T3 Ferric citrate, 5,000 mg/kg	90.38	18.64	4.50	5.20	1,023	88	4,400

**Notes:**

NA = Not applicable.

1 Calculated ferric citrate = % recovery of microtracer × ferric citrate dose.

#### Study C

The objective of this study was to evaluate three different ferric compounds as sources of dietary Fe for weaned piglets bred and managed under standard farm conditions. At weaning (25 days of age), 220 healthy piglets from a single weaning batch of ca. 340 piglets (Danbred × Piètrain) were used. Piglets were allocated to four treatment groups in a randomised block design: T1 - negative control; T2 - 500 mg ferric lactate (ca. 17% Fe)/kg feed; T3 - 500 mg ferric citrate (ca. 17% Fe)/kg feed; and T4 - 500 mg ferric tartrate (ca. 20% Fe)/kg feed. Treatments were balanced as far as possible for body weight (BW), litter origin, and gender in 44 mixed-sex pens (5 piglets/pen, males and females in each pen) giving 11 replicate pens per treatment. Basal prestarter and basal starter batches were produced and then split into 4 aliquots prior to adding the Fe sources to T2, T3 and T4 aliquots. Prestarter mash was fed for 14 days, then starter mash was fed for 28 days, until study end at 42 days post-weaning (66 d of age). Piglets were housed in post-weaning units, each unit with pens on either side of a central aisle with *ad libitum* access to feed (mash form) and water (drinking bowls).

The basal diets (T1 Control) contained 59.1 and 75.4 mg endogenous Fe/kg feed for starter and grower diets, respectively, and were Fe deficient compared to the recommended Fe inclusion rate for growing pigs (National Research Council (NRC), 2012). The ferric sources contained microtracers (MicroTracers, San Francisco, CA USA, at inclusion rate of 10%) as markers. Microtraced ferric sources were added to the basal diets instead of Tixosil^®^ (>97% colloidal silica). Feed samples were analysed for dry matter, crude protein, crude fat, ash and Fe using VDLUFA (1993) methods. Details of diets composition, analysed nutritional values and microtracer recovery are shown in Tables 5 and 6.

**Table 5:** Study C: composition and calculated analysis of starter (0–14 d of age) and grower diets (15–42 d of age).

Ingredients	Units	Starter diet				Grower diet			
		T1 Control	T2 Ferric lactate 500 mg/kg	T3 Ferric citrate 500 mg/kg	T4 Ferric tartrate 500 mg/kg	T1 Control	T2 Ferric lactate 500 mg/kg	T3 Ferric citrate 500 mg/kg	T4 Ferric tartrate 500 mg/kg
Soybean meal	%	27.00	27.00	27.00	27.00	26.20	26.20	26.20	26.20
Corn		21.07	21.07	21.07	21.07	28.46	28.46	28.46	28.46
Wheat		18.10	18.10	18.10	18.10	21.61	21.61	21.61	21.61
Barley		17.54	17.54	17.54	17.54	14.64	14.64	14.64	14.64
Skimmed milk powder		10.00	10.00	10.00	10.00	2.90	2.90	2.90	2.90
Soybean oil		1.70	1.70	1.70	1.70	1.70	1.70	1.70	1.70
Calcium carbonate		1.44	1.44	1.44	1.44	1.42	1.42	1.42	1.42
Premix^1^		1.22	1.22	1.22	1.22	1.20	1.20	1.20	1.20
Monocalcium phosphate		1.15	1.15	1.15	1.15	1.28	1.28	1.28	1.28
L-Lysine-HCL		0.39	0.39	0.39	0.39	0.34	0.34	0.34	0.34
DL-Methionine		0.18	0.18	0.18	0.18	0.10	0.10	0.10	0.10
L-Threonine		0.12	0.12	0.12	0.12	0.07	0.07	0.07	0.07
L-Tryptophan		0.06	0.06	0.06	0.06	0.03	0.03	0.03	0.03
Tixosil^®^ colloidal silica (>97% silicon dioxide)		0.550	0.495	0.495	0.495	0.550	0.495	0.495	0.495
Ferric citrate		0	0.055	0.055	0.055	0	0.055	0.055	0.055
TOTAL		100	100	100	100	100	100	100	100
AME_N_^2^	MJ/kg	13.43	13.43	13.43	13.43	13.26	13.26	13.26	13.26
Crude protein	%	21.45	21.45	21.45	21.45	19.93	19.93	19.93	19.93
Lysine		1.50	1.50	1.50	1.50	1.29	1.29	1.29	1.29
Methionine		0.54	0.54	0.54	0.54	0.41	0.41	0.41	0.41
Methionine/Cysteine		0.87	0.87	0.87	0.87	0.75	0.75	0.75	0.75
Tryptophan		0.31	0.31	0.31	0.31	0.25	0.25	0.25	0.25
Threonine		0.94	0.94	0.94	0.94	0.82	0.82	0.82	0.82
Valine		–	–	–	–	–	–	–	–
Arginine		–	–	–	–	–	–	–	–
Crude fibre		3.53	3.53	3.53	3.53	3.70	3.70	3.70	3.70
Crude fat		3.61	3.61	3.61	3.61	3.96	3.96	3.96	3.96
Starch		–	–	–	–	–	–	–	–
Crude ash		5.38	5.38	5.38	5.38	5.76	5.76	5.76	5.76
Calcium		0.96	0.96	0.96	0.96	0.88	0.88	0.88	0.88
Phosphorus		0.44	0.44	0.44	0.44	0.42	0.42	0.42	0.42
Digestible phosphorus		–	–	–	–	–	–	–	–
Sodium		0.22	0.22	0.22	0.22	0.20	0.20	0.20	0.20

**Notes:**

1 Contents per kg premix: 400,000 I.U. vit. A (acetate); 120,000 I.U. vit. D3; 8,000 mg vit. E (a-tocopherol acetate); 200 mg vit. K3 (MSB); 250 mg vit. B1 (mononitrate); 420 mg vit. B2 (cryst. riboflavin); 2,500 mg niacin (niacinamide); 400 mg Vit. B6 (HCl); 2,000 μg vit. B12; 25,000 μg Biotin (commercial, feed grade); 1,000 mg pantothenic acid (Ca d-pantothenate); 100 mg folic acid (cryst. commercial feed grade); 80,000 mg choline (chloride); 5,000 mg Zn (sulphate); 6,000 mg Mn (sulphate); 1,000 mg Cu (sulphate-pentahydrate); 20 mg Se (Na-selenite); 45 mg I (Ca-iodate); 130 g Na (NaCl); 55 g Mg (sulphate).

2 Metabolisable Energy, calculated as per DLG (2013).

**Table 6:** Study C: key analysed values of starter and grower feeds (as is).

Treatment	Dry matter (%)	Crude protein (%)	Crude fat (%)	Ash (%)	Fe (mg/kg)	Microtracer (% recovery)	Calculated ferric content (mg/kg feed)^1^
Starter diet (0–14 d)							
T1 Control	90.11	21.55	3.70	5.37	51.9	NA	NA
T2 Ferric lactate	90.08	21.60	3.68	5.41	148.9	114	570
T3 Ferric citrate	90.15	21.54	3.70	5.42	135.6	94	470
T4 Ferric tartrate	90.11	21.58	3.68	5.42	152.8	109	550
Grower diet (15–42 d)							
T1 Control	90.10	20.08	4.05	5.75	75.4	NA	NA
T2 Ferric lactate	90.11	20.10	4.06	5.81	173.5	99	500
T3 Ferric citrate	90.18	20.07	4.00	5.80	160.4	93	470
T4 Ferric tartrate	90.04	20.11	4.05	5.83	178.1	106	530

**Notes:**

NA = Not applicable.

1 Calculated ferric citrate = % recovery of microtracer × ferric citrate dose.

#### Study D

The objective of this study was to evaluate organic and inorganic sources of Fe as sources of dietary Fe for weaned piglets bred and managed under standard farm conditions. In this study, faecal samples were collected to determine Fe digestibility. At weaning (25 days of age), 175 healthy piglets from a single weaning batch of ca. 280 piglets (Danbred × Piètrain) were selected. Piglets had received routine Fe injections (Fe dextran, 200 mg intramuscular) on day 3 of age and creep feed (without addition of ferrous (II) sulphate) from day 8 to 24 of age, respectively. Weaned piglets were allocated to 5 dietary treatments: T1 - negative control; T2 - 85 mg Fe/kg from ferrous (II) sulphate; T3 - 85 Fe mg/kg from ferric (III) oxide; T4 - 500 mg ferric citrate/kg, supplying 85 mg Fe/kg feed; T5 - 500 mg ferric citrate/kg supplying 85 mg Fe/kg feed plus 85 mg Fe/kg feed from ferrous sulphate, (Table 7). Treatments were balanced as far as possible for body weight (BW), litter origin, and gender, and piglets were housed in 35 mixed-sex pens (5 piglets/pen, males and females in each pen, 7 pens per treatment). Fe supplements in treatments T2, T3 and T4 matched normal commercial practice (85 mg Fe/kg feed). Since some pig producers combine both inorganic and organic trace minerals, and to consider the effects of double supplementation, T5 contained ferric citrate and ferrous (II) sulphate, both supplying 85 mg Fe/kg feed. None of the diets exceeded the maximum EU permitted Fe in weaned piglet feeds. Starter mash was fed for 14 days, and grower mash was fed thereafter, until study end (42 days post-weaning). The creep feed contained 56.8 mg endogenous Fe/kg feed and the basal diets (T1 Control) contained 89.6 and 75.4 mg endogenous Fe/kg feed for starter and grower diets, respectively and were Fe deficient compared to the recommended Fe inclusion rate for growing pigs (National Research Council (NRC), 2012, Table 8). The ferric citrate used contained microtracer (MicroTracers, San Francisco, CA USA, at inclusion rate of 10%) as a marker. Microtraced ferric citrate was added to the basal diets instead of Tixosil^®^ (>97% colloidal silica). Titanium dioxide was included in the grower feed for faecal digestibility analysis. Feed samples were analysed for dry matter, crude protein, crude fat, ash and Fe using VDLUFA (1993) methods. Details of diets composition, analysed nutritional values and microtracer recovery are shown in Tables 7 and 8. Post-weaning piglets had *ad libitum* access to feed (mash form) and water (drinking bowls).

**Table 7:** Study D: composition and calculated analysis of creep feed, starter (0–14 d of age) and grower diets (15–42 d of age).

Ingredient/Nutrient	Units	Creep feed^1^	Starter diet					Grower diet				
			T1 Control (no Fe added)	T2 Ferrous (II) sulphate^2^	T3 Ferric (III) oxide^2^	T4 Ferric citrate^3^	T5 Ferric citrate + ferrous (II) sulphate^4^	T1 Control (no Fe added)	T2 Ferrous (II) sulphate^2^	T3 Ferric (III) oxide^2^	T4 Ferric citrate^3^	T5 Ferric citrate + ferrous (II) sulphate^4^
Corn	%	50.57	63.46	63.46	63.46	63.46	63.46	70.70	70.70	70.70	70.70	70.70
Soybean meal (CP 49%)		24.38	19.80	19.80	19.80	19.80	19.80	21.51	21.51	21.51	21.51	21.51
Skimmed milk powder		20.00	10.00	10.00	10.00	10.00	10.00	–	–	–	–	–
Mineral & vitamin premix*		1.20	1.20	1.20	1.20	1.20	1.20	1.20	1.20	1.20	1.20	1.20
Calcium carbonate		1.15	1.38	1.38	1.38	1.38	1.38	1.43	1.43	1.43	1.43	1.43
Monocalcium phosphate		1.10	1.40	1.40	1.40	1.40	1.40	1.60	1.60	1.60	1.60	1.60
Soybean oil		1.00	0.54	0.54	0.54	0.54	0.54	1.17	1.17	1.17	1.17	1.17
L-lysine HCL		0.30	0.60	0.60	0.60	0.60	0.60	0.57	0.57	0.57	0.57	0.57
DL-methionine		0.20	0.23	0.23	0.23	0.23	0.23	0.17	0.17	0.17	0.17	0.17
Cellulose		–	1.00	1.00	1.00	1.00	1.00	1.00	1.00	1.00	1.00	1.00
L-Threonine		0.19	0.19	0.19	0.19	0.19	0.19	0.17	0.17	0.17	0.17	0.17
L-tryptophan		0.10	0.10	0.10	0.10	0.10	0.10	0.08	0.08	0.08	0.08	0.08
Titanium (IV) dioxide		0	0	0	0	0	0	0.10	0.10	0.10	0.10	0.10
Corn starch or ferric source		0	0	0.10	0.10	0.10	0.10	0	0.10	0.10	0.10	0.10
TOTAL		100	100	100	100	100	100	100	100	100	100	100
AME_N_^**^	MJ/kg	13.91	13.42	13.42	13.42	13.42	13.42	13.40	13.40	13.40	13.40	13.40
Crude protein	%	24.00	20.00	20.00	20.00	20.00	20.00	18.00	18.00	18.00	18.00	18.00
Lysine		1.65	1.51	1.51	1.51	1.51	1.51	1.29	1.29	1.29	1.29	1.29
Methionine		–	0.57	0.57	0.57	0.57	0.57	0.44	0.44	0.44	0.44	0.44
Methionine/Cysteine		0.98	0.87	0.87	0.87	0.87	0.87	0.74	0.74	0.74	0.74	0.74
Tryptophan		0.37	0.30	0.30	0.30	0.30	0.30	0.25	0.25	0.25	0.25	0.25
Threonine		0.96	0.94	0.94	0.94	0.94	0.94	0.82	0.82	0.82	0.82	0.82
Valine		–	–	–	–	–	–	–	–	–	–	–
Arginine		–	–	–	–	–	–	–	–	–	–	–
Crude fibre		1.94	2.74	2.74	2.74	2.74	2.74	2.96	2.96	2.96	2.96	2.96
Crude fat		3.65	3.50	3.50	3.50	3.50	3.50	4.39	4.39	4.39	4.39	4.39
Starch		–	–	–	–	–	–	–	–	–	–	–
Crude ash		–	5.63	5.63	5.63	5.63	5.63	5.28	5.28	5.28	5.28	5.28
Calcium		0.96	0.95	0.95	0.95	0.95	0.95	0.88	0.88	0.88	0.88	0.88
Phosphorus		0.75	0.45	0.45	0.45	0.45	0.45	0.40	0.40	0.40	0.40	0.40
Digestible phosphorus		0.48										
Sodium		2.20	0.24	0.24	0.24	0.24	0.24	1.76	1.76	1.76	1.76	1.76
Fe source	mg/kg feed	0	0	ca. 266	266	550	550 + ca. 266	0	ca. 266	266	550	550 + ca. 266
Target added Fe	mg/kg feed	Only endogenous Fe	0	85	85	85	170	0	85	85	85	170

**Notes:**

* Contents per kg premix: 400,000 I.U. vit. A (acetate); 120,000 I.U. vit. D3; 8,000 mg vit. E (a-tocopherol acetate); 200 mg vit. K3 (MSB); 250 mg vit. B1 (mononitrate); 420 mg vit. B2 (cryst. riboflavin); 2,500 mg niacin (niacinamide); 400 mg Vit. B6 (HCl); 2,000 μg vit. B12; 25,000 μg Biotin (commercial, feed grade); 1,000 mg pantothenic acid (Ca d-pantothenate); 100 mg folic acid (cryst. commercial feed grade); 80,000 mg choline (chloride); 5,000 mg Zn (sulphate); 6,000 mg Mn (sulphate); 1,000 mg Cu (sulphate-pentahydrate); 20 mg Se (Na-selenite); 45 mg I (Ca-iodate); 130 g Na (NaCl); 55 g Mg (sulphate).

** Metabolisable Energy, calculated as per DLG (2013).

1 Pre-weaning creep feed with no added Fe.

2 Fe added as per commercial practice, 85 mg Fe/kg feed.

3 500 mg ferrous citrate plus 50 mg microtracer per kg feed, supplying 85 mg Fe/kg feed.

4 85 mg Fe/kg feed from ferrous citrate + 85 Fe mg/kg feed from ferrous (II) sulfate.

**Table 8:** Study D: key analysed values of creep, starter and grower feeds (as is).

Treatment	Dry matter (%)	Crude protein (%)	Crude fat (%)	Ash (%)	Fe (mg/kg)	Microtracer (% recovery)	Calculated ferric citrate content (mg/kg feed)^1^
Creep feed	92.43	24.92	3.38	6.27	56.8	NA	NA
Starter diet (0–14 d)							
T1 Control	91.09	20.09	3.60	5.48	89.6	NA	NA
T2 Fe (II) sulphate	90.96	20.05	3.64	5.52	181.7	NA	NA
T3 Fe (III) oxide	90.83	20.10	3.66	5.50	188.8	NA	NA
T4 Ferric citrate	91.22	20.18	3.62	5.53	179.6	80%	400
T5 Ferric citrate + Fe (II) sulphate	91.18	20.15	3.59	5.57	266.6	79%	395
Grower diet (15–42 d)							
T1 Control	90.98	18.62	4.47	5.40	75.4	NA	NA
T2 Fe (II) sulphate	90.86	18.62	4.48	5.39	171.5	NA	NA
T3 Fe (III) oxide	90.91	18.64	4.50	5.42	176.8	NA	NA
T4 ferric citrate	91.11	18.59	4.48	5.39	177.6	95%	475
T5 Ferric citrate + Fe (II) sulphate	90.83	18.64	4.51	5.42	256.7	88%	440

**Notes:**

NA = Not applicable.

1 Calculated ferric citrate = % recovery of microtracer × ferric citrate dose.

For all studies, temperature, relative humidity, lighting and forced ventilation were controlled and followed the breeder recommendations. All study personnel involved in taking measurements were blinded to treatments, while the study director had full knowledge of the study design.

In all studies, the general health status of the animals was monitored daily and culls and mortality recorded. During the daily health checks, if any animal was identified as showing signs of ill health or suffering, they were either culled immediately whereas if the animal was exhibiting symptoms that were potentially treatable, they were given appropriate medication. Any animal that was found dead during the daily rounds, was removed from the pen and examined. The cause of death was established and attributed to either sudden death or non-response to treatment. At study end, all surviving animals were examined by a veterinarian, fed the control diet for a week and, afterwards, returned to the farm of origin. All left-over feeds, test items and animal carcasses were disposed of according to local regulations at study end.

### Haematological and biochemical analyses (Study A, Study B and Study D)

In Study A, blood samples were collected on 35 d (study end), from two birds/pen with body weight (BW) closest to the treatment mean. For Study B and Study D, blood samples were collected on day 42 (Study B) and on day 0 and 42 (Study D) from one piglet/pen with body weight (BW) closest to the treatment mean.

In all studies, samples were collected from the anterior *vena cava* into different tubes: plain, heparinised for clinical chemistry, and EDTA tubes for blood counts.

Haematology parameters measured were erythrocytes, leukocytes, haemogram: lymphocytes, monocytes, eosinophils, basophils, neutrophils, haemoglobin, haematocrit, mean corpuscular volume–MCV, mean corpuscular haemoglobin–MCH, mean corpuscular haemoglobin concentration–MCHC and electrolyte analyses (sodium, potassium, chloride, calcium, magnesium, phosphate). Biochemistry analyses included total cholesterol, triglycerides, bilirubin, creatine, urea, glucose, albumins, globulins, total protein, enzymes (α-amylase, aspartate-amino-transferase -AST, alanine aminotransferase–ALT, glutamate-dehydrogenase–GLDH, alkaline phosphatase–AP, L-lactate dehydrogenase–LDHA), and serum amyloid A.

For Study D, blood serum was additionally analysed for Fe, superoxide dismutase (SOD) and haptoglobin. SOD is an enzyme that eliminates superoxide radicals and is an important indicator of antioxidation and oxidation levels caused by free radicals created by unbound Fe. Haptoglobin is an acute phase protein that binds to free haem and reduces oxidative activity. Blood cells were measured by flow cytometry, blood concentrations in plasma were determined by using photometry, while sodium, potassium, and chlorides were measured by ionic liquid-polyacrylamide gel electrophoresis.

### Zootechnical parameters

Standard zootechnical parameters (body weight, BW; feed intake, FI; weight gain, WG; mortality-corrected feed conversation rate, MFCR) were recorded in all studies for the different study periods.

### Fe digestibility (Study D)

On day 42, the apparent total digestibility of crude ash and Fe was determined using titanium (IV) dioxide added at a rate of 3 g/kg feed as an inert indigestible marker. A total of seven replicates from each treatment (pooled faeces collected rectally from 5 piglets per pen, giving a total of seven replicates) were collected.

To calculate the apparent total Fe digestibility, the following formula was used:

$${\rm{Total digestibility}}({\rm{\% }}) = 100 - \left( {{{{\rm{\%  Marker in feed}}} \over {{\rm{\%  Nutrient in faeces}}}} \times {{{\rm{\%  Nutrient in faeces}}} \over {{\rm{\%  Nutrient in feed}}}}} \right) \times 100$$

### Statistical Analyses

Study A and Study B were based on demonstrating non-inferiority, where the tolerance group (T3) does not perform worse than the untreated control (T1). The null hypothesis being that the animals fed the tolerance dose perform worse that the control animals. The alternative hypothesis was that the animals fed the tolerance dose perform the same or not worse than the animals fed the control diet.

Study C and Study D were based on demonstrating superiority, where the animals fed diets supplemented with ferric citrate or another source of Fe, perform better than the untreated control. The null hypothesis being that the animals fed diets containing ferric citrate (or another source of Fe) perform the same as the control animals. The alternative hypothesis being that the animals fed ferric citrate (or another source of Fe) perform better than the animals fed the control diet.

For the different studies, sample size was calculated according to the hypothesis (Sealed Envelope Ltd, 2012; primary outcome measure = BW), and were designed to be optimal or near optimal in the trade-off between power and resources.

For all studies, the basic study design was a random complete block design, with pen as the experimental unit for statistical purposes for zootechnical and faecal parameters. The individual animal was used as the experimental unit for blood parameters. All parameters were analysed using a general lineal model as follows: Y_ij_ = μ + treatment_i_ + block_j_ + ε_ij_, where Y_ij_ was the dependent variable, μ was the overall mean, treatment_i_ was the effect of treatment (Study A = 3 levels; Study B = 3 levels; Study C = 4 levels and Study D = 5 levels), block_j_ was the effect of the block, and ε_ij_ was the residual error. All experimental units were used for statistical analysis.

All parameters were reported as group least squares mean. Standard error of the mean were also reported. Multiple comparisons between treatment groups were made by Tukey test and significant differences were declared at P ≤ 0.05, while near significant trends were considered for 0.05 < *P* ≤ 0.10.

The analyses were performed using the software packages SPSS (IBM SPSS, Version 21, Study A, Study B, Study D) and Unistat (Unistat Ltd., version 10.05, Study D).

## Results

General health in all studies was good. Overall mortality rates were as follows: study A = 2.5%; study B = none; study C = 0.9%; study D = 1.14%. Details of the veterinary treatments administered are given below.

In studies A and B no antibiotics or any type of medication (other than routine vaccinations) were administered to animals. In Study C, during day 1–14, piglets were treated with amoxicillin (Hostamox® Injectable, MSD) for diarrhoea and enrofloxacin (Baytril® 100 Injectable; Bayer, Leverkusen, Germany) for purulent funiculitis and swollen joints as follows: T1 = 5 piglets for diarrhoea and 2 piglets for purulent funiculitis; T2 = 2 piglets for diarrhoea and 2 piglets for purulent funiculitis; T3 = 1 piglet for purulent funiculitis; T4 = 1 piglet for diarrhoea and 1 piglet for purulent funiculitis. Between days 15–42, animals were treated for the following reasons: T1 = 1 piglet for swollen joints; T2 = 1 piglet for swollen joints; T4 = 2 piglets for swollen joints. In Study D, amoxicillin was administered to piglets with diarrhoea between day 1 and day 22 on trial (T1 = 1; T2 = 2), to piglets with purulent funiculitis between day 1 and day 21 on trial (T1 = 3; T2 = 1; T4 = 1), and to piglets with swollen joints between day 22 and day 42 on trial (T1 = 1; T2 = 1; T3 = 1; T5 = 1). Amoxicillin was administered to individual animals on one occasion, while enrofloxacin was administered to individual animals for 3 days.

### Study A

The results summarising the safety of ferric citrate on male broiler performance are presented in Table 9. For the overall study period (0–35 days), broilers receiving the T2 (200 mg ferric citrate/kg) and T3 (2,000 mg ferric citrate/kg) diets significantly outperformed the T1 Control group in terms of BW (T2 + 4.31%; T3 + 3.32%), ADG (T2 + 4.30%; T3 + 3.34%) and MFCR (T2 - 3.26%; T3 - 3.77%). Overall mortality rate was 2.5%, indicating good bird health. Blood data from samples taken at 35 days (study end) are presented in Table 9. No significant differences were observed between the birds fed ferric citrate and birds fed the Control diet for any blood parameters measured except for erythrocytes, MCV, sodium, potassium, chloride, urea and glucose. Erythrocytes were significantly lower in T3 birds (ferric citrate, 200 mg/kg) compared to T1 Control birds (−5.04%) and MCV was significantly higher in birds fed the T3 diet (ferric citrate 2,000 mg/kg) compared to birds fed the T1 Control (+2.75%). The minerals sodium and chloride were significantly higher in birds fed T2 (ferric citrate, 200 mg/kg) and T3 (ferric citrate, 2,000 mg/kg) compared to birds fed T1 Control diet (sodium = T2 + 3.13%; T3 + 2.24%; chloride = T2 + 2.66%; T3 + 2.15%). Potassium was significantly lower in T2 birds (ferric citrate, 200 mg/kg) compared to T1 Controls (−8.98%), while urea and glucose were significantly higher in T2 birds (ferric citrate, 200 mg/kg) compared to T1 (urea = +7.5%; glucose = +4.13%).

**Table 9:** Study A: results from safety study of ferric citrate on male broilers.

Parameter	T1 Control	T2 Ferric citrate, 200 mg/kg feed	T3 Ferric citrate, 2,000 mg/kg feed	SEM	*P*-value
Performance parameters					
BW at start (g)	43.0	42.8	42.9	0.194	0.880
BW at D 14, g	429.9^a^	448.6^b^	435.0^a^	2.342	**0.001**
ADG D 0 - D 14 d (g/d)	27.6^a^	29.0^b^	28.0^a^	0.167	**0.001**
ADG D 15 - D 35 (g/d)	86.0^a^	89.7^b^	89.3^b^	0.460	**0.001**
ADFI D 0 - D 14 (g/d)	34.1	34.8	33.6	0.235	0.120
ADFI D 15 - D 35 (g/d)	121.0	122.0	120.7	0.455	0.484
MFCR D 0 - D 14	1.237	1.201	1.202	0.010	0.259
MFCR D 15 - D 35	1.408^b^	1.360^a^	1.351^a^	0.008	**0.005**
*Overall period*					
BW at D 35, g	2,236.0^a^	2,332.4^b^	2,310.3^b^	11.028	**0.001**
ADG D 0 - D 35 (g/d)	62.7^a^	65.4^b^	64.8^b^	0.316	**0.001**
ADFI D 0 - D 35 (g/d)	86.3	87.1	85.8	0.326	0.280
MFCR D 0 - D 35	1.377^b^	1.332^a^	1.325^a^	0.007	**0.003**
Blood parameters at 35 days					
Erythrocytes (T/l)	2.38^b^	2.33^ab^	2.26^a^	0.016	**0.003**
Leukocytes (G/l)	8.78	8.96	9.39	0.295	0.699
Lymphocytes (%)	52.89	52.50	54.19	1.129	0.821
Heterophils (%)	30.95	31.45	28.90	0.946	0.516
Monocytes (%)	7.07	7.42	7.58	0.366	0.848
Eosinophils (%)	2.90	4.24	3.88	0.237	0.053
Basophils (%)	6.20	4.39	5.44	0.342	0.095
Haemoglobin (g/l)	94.5	93.56	89.94	0.877	0.078
Haematocrit (l/l)	0.24	0.24	0.24	0.002	0.295
MCV^1^ (Fl)	101.63^a^	103.38^ab^	104.37^b^	0.383	**0.010**
MCH^2^ (Pg)	39.66	40.11	39.80	0.226	0.723
MCHC^3^ (g/dl)	39.19	38.79	38.17	0.218	0.160
Sodium (mmol/l)	149.44^a^	154.13^b^	152.8^b^	0.446	**<0.001**
Potassium (mmol/l)	5.34^b^	4.86^a^	4.94^ab^	0.085	**0.042**
Chloride (mmol/l)	110.25^a^	113.19^b^	112.63^b^	0.335	**<0.001**
Calcium (mmol/l)	2.76	2.80	2.76	0.015	0.445
Magnesium (mmol/l)	1.02	1.08	1.04	0.011	0.115
Phosphate (mmol/l)	2.57	2.69	2.64	0.023	0.093
α-Amylase (U/l)	603	667	634	23.138	0.543
AST^4^ (U/l)	256	293	287	15.216	0.565
ALT^5^ (U/l)	3.1	3.3	3.1	0.064	0.397
GLDH^6^ (U/l)	2.0	2.1	2.1	0.035	0.315
ALP^7^ (U/l)	15,617	13,503	1,194	887.823	0.126
LDHA^8^ (U/l)	1,203	1,389	1,365	119.718	0.795
CK^9^ (U/l)	11,447	10,866	11,385	1049.53	0.971
Total cholesterol (mmol/l)	3.40	3.52	3.26	0.049	0.093
Triglycerides (mmol/l)	1.87	1.62	1.89	0.054	0.065
Urea (mmol/l)	0.80^a^	0.86^b^	0.82^ab^	0.011	**0.043**
Total bilirubin (µmol/l)	1.83	1.83	1.65	0.039	0.090
Creatine (µmol/l)	6.73	7.64	7.05	0.232	0.276
Glucose (mmol/l)	14.03^a^	14.61^b^	14.51^ab^	0.095	**0.025**
Albumins (mmol/l)	13.40	13.56	12.79	0.280	0.506
Globulins (mmol/l)	17.73	17.94	18.03	0.404	0.954
Total protein (mmol/l)	31.13	31.50	30.81	0.299	0.653
Serum Amyloid A (mg/l)	<5	<5	<5	–	**-**

**Notes:**

D, day; BW, average body weight (g/bird); ADG, average daily weight gain (g/bird/day); ADFI, average daily feed intake (g/bird/day); MFCR, feed conversion ratio (feed/gain adjusted for mortality/culls).

^abc^Values in same row with different superscript are significantly different (*P* ≤ 0.05).

*P*-values in bold are *P* ≤ 0.05.

Values are least square means of 16 birds/treatment (2 birds/pen and 8 pen/treatment).

SEM, Standard Error of the Mean.

1 Mean corpuscular volume.

2 Mean corpuscular haemoglobin.

3 Mean corpuscular haemoglobin concentration.

4 Aspartate transaminase.

5 Alanine aminotransferase.

6 Glutamate dehydrogenase.

7 Alkaline phosphatase.

8 L-lactate dehydrogenase.

9 Creatine kinase.

### Study B

Results summarising the safety of ferric citrate in weaned piglets are presented in Table 10. For the overall study period (0–42 days) there were no significant differences between treatment groups for BW, ADG or ADFI. However, FCR was significantly improved in piglets fed T2 (ferric citrate, 500 mg/kg) and T3 (ferric citrate, 5,000 mg/kg) diets compared to those fed the T1 Control diet (T2 = −4.02%; T3 = −5.31%). There were no significant differences between treatment groups for any of the blood parameters measured.

**Table 10:** Study B: results from safety study of ferric citrate on weaned piglets.

Parameter	T1 Control	T2 Ferric citrate, 500 mg/kg feed	T3 Ferric citrate, 5,000 mg/kg feed	SEM	*P*-value
Performance parameters					
Mean age at weaning (d)	24.94	25.00	25.00	0.140	0.980
N° replicate pens post-weaning	08	08	08		
N° piglets per pen at weaning	06	06	06		
Mean BW at weaning (kg)	7.35	7.35	7.36	0.042	0.999
Mean BW at 14 d post-weaning (kg)	9.48	9.66	9.84	0.082	0.217
Mean BW at 42 d post-weaning (kg)	24.50^x^	25.21^xy^	25.47^y^	0.184	0.075
ADG 1–14 d post-weaning (g)	152	165	177	5.112	0.138
ADG 15–42 d post-weaning (g)	536	555	558	4.726	0.114
ADG 1–42 d post-weaning (g)	408^x^	425^xy^	431^y^	4.257	0.066
ADFI 1–14 d post-weaning (g)	218	225	232	6.978	0.737
ADFI 15–42 d post-weaning (g)	835	832	829	6.504	0.919
ADFI 1–42 d post-weaning (g)	630	630	630	5.627	1.000
FCR 1–14 days post-weaning (feed:gain)	1.426^a^	1.368^ab^	1.304^b^	0.090	**0.017**
FCR 15–42 d post-weaning (feed:gain)	1.559^a^	1.499^b^	1.484^b^	0.010	**0.001**
FCR 1–42 d post-weaning (feed:gain)	1.542^a^	1.48^b^	1.460^b^	0.010	**0.001**
Blood parameters					
Erythrocytes (T/l)	6.39	6.09	6.46	0.098	0.271
Leukocytes (G/l)	17.05	16.46	16.89	0.808	0.958
Thrombocytes (G/l)	485.88	423.38	461.63	24.440	0.595
Lymphocytes (%)	51.75	48.88	57.38	2.198	0.287
Neutrophils (%)	38.75	41.63	33.75	2.293	0.382
Monocytes (%)	5.75	4.38	4.75	0.397	0.359
Eosinophils (%)	1.75	2.38	2.13	0.169	0.331
Basophils (%)	0.88	0.75	0.88	0.130	0.910
Other cells (%)	1.25	2.00	1.50	0.248	0.472
Haemoglobin (g/l)	104.75	105.63	110.00	1.933	0.514
Haematocrit (l/l)	0.34	0.33	0.35	0.006	0.475
MCV^1^ (Fl)	52.54	53.80	54.03	0.627	0.601
MCH^2^ (Pg)	16.40	17.33	17.08	0.222	0.218
MCHC^3^ (g/dl)	31.23^x^	32.23^y^	31.59^xy^	0.183	0.073
Sodium (mmol/l)	144.75	143.75	144.75	0.458	0.610
Potassium (mmol/l)	6.08	5.73	5.73	0.133	0.485
Chloride (mmol/l)	104.13	103.00	103.88	0.389	0.483
Calcium (mmol/l)	2.62	2.62	2.73	0.024	0.129
Magnesium (mmol/l)	0.91	0.94	0.92	0.016	0.791
Phosphate (mmol/l)	3.22	3.35	3.25	0.050	0.592
α-Amylase (U/l)	3382.25	2931.50	2928.50	116.206	0.190
AST^4^ (U/l)	47.25	46.38	45.25	2.673	0.958
ALT^5^ (U/l)	64.13^xy^	65.75^x^	54.75^y^	2.186	0.080
GLDH^6^ (/U/l)	2.07	2.33	2.11	0.114	0.627
AP^7^ (U/l)	329.88	367.13	303.13	13.684	0.160
LDHA^8^ (U/l)	765.38	699.38	720.13	37.196	0.776
CK^9^ (U/l)	1378.50	1087.13	1580.13	158.582	0.462
Total cholesterol (mmol/l)	1.71	1.82	1.69	0.048	0.523
Triglycerides (mmol/l)	0.40	0.46	0.42	0.030	0.710
Urea (mmol/l)	1.54	1.72	1.95	0.107	0.312
Total bilirubin (µmol/l)	1.22	1.51	1.54	0.259	0.470
Creatine (µmol/l)	69.73	65.05	67.69	1.975	0.645
Glucose (mmol/l)	5.88	5.80	6.00	0.070	0.512
Albumins (mmol/l)	25.69	26.20	26.58	0.475	0.762
Globulins^10^ (mmol/l)	20.69	20.18	21.30	0.544	0.718
Total protein (mmol/l)	46.38	46.38	47.88	0.566	0.478
Haptoglobin (mg/ml)	0.55	0.45	0.44	0.032	0.309

**Notes:**

Values are least square means of 8 pens/treatment (6 piglets/pen).

SEM, Standard Error of the Mean; BW, body weight; ADG, average daily weight gain; ADFI, average daily feed intake; FCR, feed conversion ratio.

1 Mean corpuscular volume.

2 Mean corpuscular haemoglobin.

3 Mean corpuscular haemoglobin concentration.

4 Aspartate transaminase.

5 Alanine aminotransferase.

6 Glutamate dehydrogenase.

7 Alkaline phosphatase.

8 L-lactate dehydrogenase.

9 Creatine kinase.

10 Amount of Globulins is calculated by subtracting the amount of Albumins from Total protein.

Different superscripts in same row are significant or trending (a/b: *P* ≤ 0.05; x/y 0.05 < *P* ≤ 0.10).

*P*-values in bold are *P* ≤ 0.05.

### Study C

Results summarising the effects of ferric citrate and other ferric Fe sources fed to weaned piglets are presented in Table 11. For the overall study period, significant improvements were observed in groups fed the ferric Fe sources compared to those fed the control diets. On day 42, animals in T3 (ferric citrate) and T4 (ferric tartrate) were 1.41 kg and 1.45 kg heavier, respectively compared to the T1 Control animals. Furthermore, animals fed T3 and T4 diets grew significantly better than T1 Controls (ADG: T3 = +7.09%; T4 = +7.30%). FCR was also significantly improved in animals fed T3 and T4 compared to those fed T2 (ferric lactate) (T3 = −3.84%; T4 = −3.22%) and the T1 Controls (T3 = −8.36%;T4 = −7.77%).

**Table 11:** Study C: results comparing different iron sources fed to weaned piglets from weaning for 42 days.

Parameter	T1 Control	T2 Ferric lactate, 500 mg/kg feed	T3 Ferric citrate, 500 mg/kg feed	T4 Ferric tartrate, 500 mg/kg feed	SEM	*P*-value
Mean BW at weaning (kg)	7.70	7.70	7.69	7.69	0.138	1.000
Mean BW at 14 d post-weaning (kg)	10.52	10.83	10.97	10.77	0.114	0.582
Mean BW at 42 d post-weaning (kg)	27.82^a^	28.68^ab^	29.23^b^	29.27^b^	0.156	**0.001**
ADG 1–14 d post-weaning (g)	201	224	235	220	0.005	0.131
ADG 15–42 d post-weaning (g)	618^a^	637^ab^	652^b^	661^b^	0.005	**0.004**
ADG 1–42 d post-weaning (g)	479^a^	500^ab^	513^b^	514^b^	0.004	**0.002**
ADFI 1–14 d post-weaning (g)	266	275	288	282	0.006	0.641
ADFI 15–42 d post-weaning (g)	965	955	935	945	0.006	0.294
ADFI 1–42 d post-weaning (g)	732	728	719	724	0.004	0.760
FCR 1–14 days post-weaning (feed:gain)	1.327^b^	1.229^ab^	1.226^a^	1.283^ab^	0.014	**0.030**
FCR 15–42 d post-weaning (feed:gain)	1.564^c^	1.499^b^	1.433^a^	1.433^a^	0.010	**<0.001**
FCR 1–42 d post-weaning (feed:gain)	1.530^c^	1.458^b^	1.402^a^	1.411^a^	0.010	**<0.001**

**Notes:**

N° replicates, 11; SEM, Standard error of mean; BW, body weight; ADG, average daily gain; ADFI, average daily feed intake; FCR, feed conversion ratio.

Values in same column with no common superscript are significantly different (a,b,c: *P* ≤ 0.05).

*P*-values in bold are *P* ≤ 0.05.

### Study D

Results summarising the effects of ferric citrate and other sources of Fe fed to weaned piglets are presented in Table 12. On day 42 piglets fed T4 (ferric citrate) and T5 (ferric citrate + Fe (II) sulphate) weighed significantly more compared to piglets fed T3 (Fe (III) oxide) (+0.81 kg and +0.77 kg, respectively), T2 (Fe (II) sulphate, +0.95 kg and +0.91 kg, respectively) and T1 (Control, +1.53 kg and +1.49 kg, respectively). Piglets in T4 and T5 also grew significantly better and had improved FCR compared to piglets fed T2 and T1 diets (ADG: T4 *vs*. T2 = +5.63%; T4 *vs*. T1 = +9.39%; T5 *vs*. T2 = +5.39%; T5 *vs*. T1 = +9.13%; FCR: T4 *vs*. T2 = −4.13%; T4 *vs*. T1 = −6.93%; T5 *vs*. T2 = −4.41%; T5 *vs*. T1 = −7.20%). Faecal ash digestibility was significantly better in all treatment groups compared to the T1 Control (T2 = +15.27%; T3 = +19.43%; T4 = +14.27%; T5 = +16.80%) and Fe digestibility was significantly improved in piglets fed T4 (ferric citrate, +96.71%) and T5 (ferric citrate + Fe (II) sulphate, +70.84%) compared to the T1 Control animals. Fe content in blood serum was not significantly different between treatment groups at either 25 or 66 days of age.

**Table 12:** Study D: results comparing different iron sources fed to weaned piglets from weaning for 42 days.

Parameter	T1 Control	T2 Fe (II) sulphate, 85 mg Fe/kg feed	T3 Fe (III) oxide, 85 mg Fe/kg feed	T4 Ferric citrate, 85 mg Fe/kg feed	T5 Ferric citrate + Fe (II) sulphate, 170 mg Fe/kg feed	SEM	*P*-value
Performance parameters							
Mean age at weaning (d)	24.77	24.69	24.66	24.77	24.74		
N° replicate pens post-weaning	07	07	07	07	07		
N° piglets per pen at weaning	05	05	05	05	05		
Mean BW at weaning (kg)	6.16	6.16	6.13	6.17	6.16	0.049	1.000
Mean BW at 14 d post-weaning (kg)	7.86	8.04	8.07	8.34	8.31	0.061	0.218
Mean BW at 42 d post-weaning (kg)	22.73^a^	23.31^ab^	23.45^b^	24.26^c^	24.22^c^	0.103	**<0.001**
ADG 1–14 d post-weaning (g)	121^a^	134^ab^	137^ab^	156^b^	153^b^	2.975	**0.004**
ADG 15–42 d post-weaning (g)	531^a^	545^ab^	549^ab^	569^b^	568^b^	3.498	**0.004**
ADG 1–42 d post-weaning (g)	394^a^	408^ab^	412^abc^	431^c^	430^c^	2.646	**<0.001**
ADFI 1–14 d post-weaning (g)	152	164	160	174	171	3.296	0.460
ADFI 15–42 d post-weaning (g)	794	793	794	798	795	4.746	0.998
ADFI 1–42 d post-weaning (g)	580	583	583	590	587	3.351	0.966
FCR 1–14 days post-weaning (feed:gain)	1.252^x^	1.219^xy^	1.175^xy^	1.122^y^	1.114^y^	0.015	0.061
FCR 15–42 d post-weaning (feed:gain)	1.497^a^	1.453^ab^	1.446^ab^	1.403^b^	1.399^b^	0.008	**<0.001**
FCR 1–42 d post-weaning (feed:gain)	1.471^c^	1.428^bc^	1.415^abc^	1.369^ab^	1.365^a^	0.010	**<0.001**
Faecal digestibility of iron on day 42							
Mean faecal dry matter (%)	36.32	37.52	36.20	39.31	39.10	0.444	0.147
Mean apparent total-tract digestibility of ash (%)	45.04^a^	51.92^b^	53.82^b^	51.47^b^	52.61^b^	0.659	**<0.001**
Mean apparent total-tract digestibility of iron (%)	10.05^a^	12.59^ab^	13.69^ab^	19.77^c^	17.17^bc^	0.781	**<0.001**
Iron content in blood serum on day 0 and 42							
Iron content d 25 of age (mg/l))	0.99	0.97	0.98	0.96	0.98	0.017	0.999
Iron content d 66 of age (mg/l)	1.09	1.13	1.14	1.16	1.16	0.010	0.246

**Notes:**

Values are least square means of 5 piglets/pen.

SEM, Standard Error of the Mean; BW, body weight; ADG, average daily weight gain; ADFI, average daily feed intake; FCR, feed conversion ratio.

Different superscripts in same row are significant or trending (a/b/c: *P* ≤ 0.05; x/y 0.05 < *P* ≤ 0.10).

*P*-values in bold are *P* ≤ 0.05.

The effects of ferric citrate and other sources of Fe on blood parameters of weaned piglets are presented in Tables 13 and 14. On day 0 there were no significant differences in blood parameters between treatment groups. On day 42, no significant differences were observed for any of the blood parameters except for total bilirubin and SOD. Total bilirubin was significantly higher in T2 compared to the other treatment groups (T1 = +74.73%; T3 = +63.54%; T4 = +78.49%; T5 = +95.29%), while SOD was significantly higher in the animals fed ferric citrate (T4 and T5) compared to the piglets fed the T1 Control diet (T4 = +33.73%; T5 = +29.51%), T2 (T4 = +35.36%; T5 = +31.09%) and T3 (T4 = +30.58%; T5 = +26.47%).

**Table 13:** Study D: blood analyses on day 0.

Parameter	T1 Control	T2 Fe (II) sulphate, 85 mg Fe/kg feed	T3 Fe (III) oxide, 85 mg Fe/kg feed	T4 Ferric citrate, 85 mg Fe/kg feed	T5 Ferric citrate + Fe (II) sulphate, 170 mg Fe/kg feed	SEM	*P*-value
Erythrocytes (T/l)	5.87	5.97	5.86	5.98	6.06	0.084	0.985
Leukocytes (G/l)	9.95	9.41	9.16	9.20	9.13	0.315	0.980
Thrombocytes (G/l)	246.1	261.6	255.6	251.9	247.9	10.910	0.975
Lymphocytes (%)	46.86	48.86	43.00	45.57	47.43	0.614	0.113
Neutrophils (%)	44.71	43.86	48.29	46.43	44.86	0.609	0.367
Monocytes (%)	3.57	3.00	3.57	3.14	3.43	0.241	0.961
Eosinophils (%)	3.57	3.14	3.86	3.29	2.71	0.257	0.854
Basophils (%)	0.43	0.71	0.57	0.57	1.00	0.539	0.361
Other cells (%)	0.86	0.43	0.71	1.00	0.57	0.111	0.623
Haemoglobin (g/l)	100	101	100	102	101	0.478	0.946
Haematocrit (l/l)	0.33	0.35	0.35	0.35	0.35	0.003	0.282
MCV^1^ (Fl)	57.19	58.48	60.76	59.32	58.56	1.011	0.864
MCH^2^ (Pg)	17.22	17.02	17.27	17.11	16.82	0.249	0.997
MCHC^3^ (g/dl)	29.74	29.18	28.60	29.09	29.09	0.302	0.734
Sodium (mmol/l)	140	139	141	139	140	0.373	0.795
Potassium (mmol/l)	5.0	5.0	4.9	4.8	5.0	0.067	0.981
Chloride (mmol/l)	101	102	101	101	100	0.266	0.826
Calcium (mmol/l)	2.61	2.58	2.62	2.59	2.60	0.025	0.999
Phosphate (mmol/l)	3.31	3.27	3.29	3.26	3.25	0.035	0.997
AST^4^ (U/l)	38	36	37	38	36	1.041	0.989
ALT^5^ (U/l)	42	43	44	43	42	1.029	0.993
GLDH^6^ (/U/l)	<2	<2	<2	<2	<2		
AP^7^ (U/l)	633	602	627	585	590	22.855	0.990
Total cholesterol (mmol/l)	3.44	3.37	3.49	3.32	3.35	0.058	0.958
Triglycerides (mmol/l)	0.93	0.95	0.99	0.95	0.93	0.040	0.991
Urea (mmol/l)	3.09	3.12	3.05	2.99	2.93	0.090	0.991
Total bilirubin (µmol/l)	11.0	10.5	11.1	10.5	11.4	0.365	0.894
Glucose (mmol/l)	4.01	3.94	3.83	3.91	3.90	0.083	0.993
Albumins (mmol/l)	30.4	30.0	29.1	29.7	29.9	0.296	0.880
Total protein (mmol/l)	51	51	50	49	49	0.554	0.779
Haptoglobin (mg/ml)	0.60	0.55	0.74	0.61	0.51	0.063	0.158
SOD^8^ (U/ml)	1.27	1.24	1.25	1.25	1.27	0.026	0.999
SOD^8^ (U/g Haemoglobin)	1,272	1,023	1,242	1,228	1,250	25.805	0.998

**Notes:**

Values are means of 1 piglet/pen.

SEM, Standard Error of the Mean.

1 Mean corpuscular volume.

2 Mean corpuscular haemoglobin.

3 Mean corpuscular haemoglobin concentration.

4 Aspartate transaminase.

5 Alanine aminotransferase.

6 Glutamate dehydrogenase.

7 Alkaline phosphatase.

8 Superoxide dismutase.

**Table 14:** Study D: blood analyses on day 42.

Parameter	T1 Control	T2 Fe (II) sulphate, 85 mg Fe/kg feed	T3 Fe (III) oxide, 85 mg Fe/kg feed	T4 Ferric citrate, 85 mg Fe/kg feed	T5 Ferric citrate + Fe (II) sulphate, 170 mg Fe/kg feed	SEM	*P*-value
Erythrocytes (T/l)	6.62	6.42	6.51	6.38	6.49	0.087	0.915
Leukocytes (G/l)	16.41	16.03	17.79	17.23	19.03	0.672	0.741
Thrombocytes (G/l)	502	537	532	587	542	25.901	0.725
Lymphocytes (%)	44.57	43.57	44.57	40.86	45.71	1.079	0.854
Neutrophils (%)	46.14	46.14	45.57	46.86	44.57	1.110	0.987
Monocytes (%)	3.71^xy^	3.29^x^	5.14^xy^	5.86^y^	4.43^xy^	0.290	0.053
Eosinophils (%)	2.71	3.14	3.43	4.57	3.14	0.310	0.402
Basophiles (%)	0.71	0.71	0.86	0.57	1.00	0.067	0.557
Other cells (%)	2.14	3.14	0.29	1.29	1.14	0.311	0.115
Haemoglobin (g/l)	102	102	101	105	104	1.043	0.850
Haematocrit (l/l)	0.34	0.35	0.34	0.35	0.35	0.003	0.837
MCV^1^ (Fl)	51.76	52.60	54.59	56.26	55.96	0.758	0.437
MCH^2^ (Pg)	15.44	15.56	16.07	17.01	16.76	0.273	0.497
MCHC^3^ (g/dl)	29.83	29.30	28.81	30.21	29.91	0.219	0.480
Sodium (mmol/l)	140.6	140	142	142	143	0.562	0.559
Potassium (mmol/l)	5.64	4.86	5.27	5.41	5.61	0.115	0.103
Chloride (mmol/l)	102	101	102	104	102	0.504	0.701
Calcium (mmol/l)	2.68	2.64	2.72	2.69	2.77	0.039	0.649
Phosphate (mmol/l)	3.45	3.20	3.42	3.40	3.64	0.084	0.733
AST^4^ (U/l)	47.71	38.71	44.14	41.43	43.43	2.446	0.922
ALT^5^ (U/l)	49.86	58.00	46.42	51.00	53.29	1.993	0.690
GLDH^6^ (U/l)	2.10	2.00	3.87	1.97	1.99	0.296	0.357
AP^7^ (U/l)	263	272	297	242	239	7.329	0.205
Total cholesterol (mmol/l)	2.63	3.26	2.86	2.18	2.22	0.155	0.167
Triglycerides (mmol/l)	0.66^xy^	0.77^y^	0.48^x^	0.64^xy^	0.59^xy^	0.029	0.069
Urea (mmol/l)	2.86	2.83	2.16	2.45	2.52	0.177	0.775
Total bilirubin (µmol/l)	1.90^a^	3.32^b^	2.03^a^	1.86^a^	1.70^a^	0.145	**0.003**
Glucose (mmol/l)	5.06	5.54	4.77	4.89	5.33	0.127	0.313
Albumins (mmol/l)	27.97	30.63	27.3	28.9	27.7	0.438	0.162
Total protein (mmol/l)	51.29	50.86	50.57	52.00	52.57	0.564	0.911
Haptoglobin (mg/ml)	1.07	1.05	1.01	0.98	0.96	0.017	0.198
SOD^8^ (U/ml)	1.66^a^	1.64^a^	1.70^ab^	2.22^c^	2.15^c^	0.048	**<0.001**
SOD^8^ (U/g Haemoglobin)	1,641^a^	1,611^a^	1,685^ab^	2,12^c^	2,068^bc^	48.060	**<0.001**

**Notes:**

Values are means of 1 piglet/pen.

SEM, Standard Error of the Mean.

1 Mean corpuscular volume.

2 Mean corpuscular haemoglobin.

3 Mean corpuscular haemoglobin concentration.

4 Aspartate transaminase.

5 Alanine aminotransferase.

6 Glutamate dehydrogenase.

7 Alkaline phosphatase.

8 Superoxide dismutase.

Different superscripts in same row are significant or trending (a/b/c: *P* ≤ 0.05; x/y 0.05 < *P* ≤ 0.10).

*P*-values in bold are *P* ≤ 0.05.

## Discussion

The objective of the four studies presented here was to assess the safety and efficacy of ferric citrate as a safe and available source of Fe for broilers and weaned piglets bred and managed under standard farming conditions.

### Safety in broilers - Study A

Haemoglobin is an Fe-rich protein involved in cellular metabolism and the transport of oxygen through animal tissues (Snyder & Sheafor, 1999). Thus, haemoglobin and haematocrit-related parameters are key to detecting any possible negative effects of dietary Fe supplementation in broilers. No significant differences between diets were detected for haemoglobin, haematocrit, MCH or MCHC. In the case of erythrocyte and MCV values, despite the statistically significant differences with the Control diet, all results were in line with values reported in previous studies (Milanovic et al., 2008; Saripinar-Aksu, Aksu & Özsoy, 2010; Tako, Rutzke & Glahn, 2010). Likewise, the statistically significant differences detected between treatments for sodium, potassium, chloride, urea and glucose were considered to have no clinical relevance since there were no indications of a dose-dependent effect and results were in agreement with other reported data for chickens (Simaraks, Chinrasri & Aengwanich, 2004; Café et al., 2012; Odunitan-Wayas, Kolanisi & Chimonyo, 2018). Furthermore, the inflammatory marker serum-amyloid A (O’Reilly, Bailey & Eckersall, 2018) was below the detection level, indicating good health status of all birds. Finally, and with the few exceptions described above, blood cells, enzymes, electrolytes and biochemical blood constituents at the end of the 35 days feeding period did not change significantly across the different doses of ferric citrate. Altogether, the analysis of blood data shows that ferric citrate is a safe source of supplementary Fe for broilers, since no negative effects were detected in either of the ferric citrate treatments, where supplementary Fe from ferric citrate supplied an additional 34 mg (T2) and 340 mg (T3) Fe/kg feed, approximately, in comparison with T1 Control (only endogenous dietary Fe). Additionally, it was confirmed that ferric citrate is well tolerated up to 2,000 mg/kg (×10 the recommended inclusion rate). These results are in line with a recent assessment performed by the European Food Safety Authority (EFSA, 2019), who concluded that ferric citrate is a safe feed additive for weaned piglets up to 5,000 mg/kg of feed, (supplying ca. 850 mg Fe/kg feed).

As indicated by the National Research Council (NRC) (1994), some of the response criteria for Fe supplementation in chickens are growth and feed efficiency. The results presented in Study A support this as, after 35 days, animals fed diets supplemented with ferric citrate at either 200 mg/kg or 2,000 mg/kg feed (34 and 340 mg Fe/kg feed, respectively) showed better performance in terms of ADG, MFCR and final weight compared to birds with no Fe supplementation (T1 Control = ${\rm{\bar{x}}}$ 79 mg Fe/kg Fe). The low mortality rate reported (2.5% at 35 days), coupled with the blood analysis and the growth performance, support the safety of ferric citrate in broilers.

The results of Study A are in agreement with an earlier study that evaluated ferric citrate as a source of Fe, using broiler chickens in a specialised model intended as an intermediate test of *in vivo* Fe bioavailability of human Fe supplements (Tako, Rutzke & Glahn, 2010). Intestinal Fe absorption and haemoglobin maintenance was assessed in broilers using ferric citrate as the Fe source. From one week of age the control group was fed a diet deficient in Fe (51 mg Fe/kg feed), and the other group was fed a diet supplemented with 500 mg ferric citrate/kg (total Fe: 141 mg/kg feed). During the 6-week study period, there was no difference in feed intake between the treatments, but Fe intake was significantly increased in the ferric citrate supplemented group compared to the Fe deficient control group. Blood haemoglobin (Hb) concentrations were also significantly higher in the ferric citrate group compared with the control. The Hb maintenance efficiency values (indicator for dietary Fe availability) were significantly higher in birds fed the Fe-deficient control diet, indicating that the Fe uptake mechanisms in broiler are responding to dietary Fe concentrations. The final total body Hb and the increase in total body haemoglobin Fe during the study period was significantly greater in the ferric citrate supplemented group than in the control group (85 *vs*. 54 mg, *P* < 0.05). No mortalities or adverse effects were reported.

Whilst not evaluated in the present study, it has been demonstrated that organic sources of Fe are more bioavailable in broilers than inorganic forms (Pla & Fritz, 1970; Shinde et al., 2011; Arnaudova-Matey et al., 2013). For example, Pla & Fritz (1970) investigated the bioavailability of organic and inorganic sources of Fe in broiler chicks with severe anaemia. Over a two-week period, response to dietary Fe supplementation was measured using blood Hb. The authors categorized the sources of Fe into good, mediocre and poor. They reported that the organic sources of iron, including ferric citrate, were good sources of iron compared to ferrous sulphate. Shinde et al. (2011) looked at the efficiency of organic and inorganic sources of Fe in Fe-depleted broilers and compared ferric sulphate and ferrous methionine with a negative control diet. During 35 days, improved body weight gain, FCR, and greater dry matter, crude protein retention and Fe digestibility in faeces were observed in birds fed on Fe supplemented diets when compared with birds fed the control diet. After 35 days, the red blood cell, Hb, haematocrit, and Fe concentration in plasma, tibia and liver were higher in birds fed on Fe-supplemented diets than birds fed on the control diet. Most importantly, supplementation of Fe in the organic form resulted in greater Fe concentration in the tibia and liver, and less Fe excretion, when compared with birds receiving inorganic Fe.

### Safety in weaned piglets

The results obtained in Study B support the safety of ferric citrate. In this study, no adverse effects on growth or blood parameters were noted. Furthermore, no mortality was recorded, and animals were healthy for the duration of the trial. These results support the safety of ferric citrate when added to the diet of weaned piglets at up to 10 times the recommended dose, and are in agreement with the recent assessment performed by the European Food Safety Authority (EFSA, 2019), who concluded that ferric citrate is a safe feed additive for weaned piglets up to 5,000 mg/kg of feed. Results obtained in Studies C and D further support the safety of ferric citrate as a source of dietary Fe. In these studies, no adverse effects were observed in the animals fed ferric citrate at the recommended dose (ferric citrate at 500 mg/kg feed supplying 85 mg Fe/kg feed); animals fed ferric citrate performed better than animals fed the control diet (containing endogenous Fe only), and the blood data were within the normal ranges for healthy piglets.

### Nutritional efficacy in weaned piglets

The results from Studies C and D showed that ferric citrate is an effective source of iron in weaned piglets. Study C demonstrated that ferric citrate and other sources of ferric Fe improved growth rates in pigs fed these compounds compared to pigs fed the Fe deficient negative control diet containing only endogenous Fe. The results from Study D showed that pigs fed ferric citrate alone or in combination with ferrous sulphate performed better than the control animals and the animals fed inorganic forms of iron. Moreover, in Study D, Fe digestibility was significantly improved in piglets fed ferric citrate alone or in combination with ferrous sulphate compared to the control animals and the animals fed the inorganic forms of Fe, thus supporting the nutritional efficacy of ferric citrate. In the same study, no significant improvements in blood Fe parameters were observed. At the beginning of the study (25 days of age), all piglets had Hb of 100 g/L to 102 g/L, which indicated normal iron concentrations and that the animals were not iron deficient or anaemic (Wei et al., 2005). At day 42 (66 days of age), piglets fed ferric citrate had numerically higher Hb compared to the negative controls, but all groups were within the expected range. No significant differences were noted for other blood parameters indicative of Fe status (MCV, MCH, MCHC), highlighting that all piglets included in this study were not seriously iron deficient or anaemic. Furthermore, no significant differences in Fe serum concentrations were seen between treatment groups on day 0 or day 42. These results are not surprising given that all piglets received a parenteral iron dextran injection at 3 days old. Administration of iron dextran is standard practice and performed for welfare reasons to reduce severe Fe deficiency and high mortality in pre- and post-weaning piglets (Perri et al., 2016; Svoboda, Vanhara & Berlinska, 2017). As these studies were run for regulatory approval, using Fe-depleted animals was not appropriate as healthy animals must be used to support the safety and efficacy of feed additives (EFSA, 2017; EFSA, 2018). However, earlier studies reported that ferric citrate supplemented at daily use rates of 48 mg and 144 mg/feed increased blood haemoglobin concentration and plasma Fe in pigs (Furugouri & Kawabata, 1975) and interstingly, Furugouri & Kawabata (1975) also cited a study performed by Ullrey et al. (1973), who reported that ferric citrate is a more effective source of supplemental Fe for piglets than ferrous sulfate.

Even though a Fe-depletion model which is often described as the standard for assessing bioavailability of iron (Henry & Miller, 1995) was not used in the present studies, improved growth performance in animals fed ferric citrate compared to the animals fed diets containing endogenous Fe only was observed. This, coupled with the faecal digestibility of Fe, indicates that ferric citrate is a safe and effective source of dietary Fe. Furthermore, piglets receiving ferric citrate had significantly higher levels of SOD compared to the negative control group and the groups fed the inorganic forms of Fe, indicating that oral administration of ferric citrate improved the oxidative status of the animals. These results were in agreement with a recent study that compared oral administration of ferrous glycine and parenteral iron dextrose with a negative control in anaemic pigs (Dong et al., 2020). These authors reported significantly decreased serum SOD levels in the control animals and those that received iron dextran compared to the animals fed the organic form of Fe.

Some animals in studies C and D were treated with antibiotics, which could raise some concern given that there are reports showing an interference of these compounds with iron absorption (Djaldetti et al., 1981). However, the data presented in study D shows that all animals had recommended Fe levels and no significant differences were found in Fe serum concentrations between treatment groups. Additionally, the proportion of animals treated in each study was low (<9% out of 220 and 175 piglets, respectively) and antibiotics were applied roughly equally across all treatment groups.

Iron requirements for piglets are influenced by several factors, the most important being growth intensity during pre-weaning and post-weaning. Modern pig breeds are selected for fast growth and several recent studies have questioned whether current Fe supplementation practices are meeting Fe requirements (Perri et al., 2016; Svoboda, Vanhara & Berlinska, 2017). Hence, dietary supplementation with Fe is required to ensure that adequate stores of Fe are achieved. Inadequate iron leads to health and economic implications as piglets with depleted iron stores are at risk of a suppressed immune system, leading to an impaired ability to resist infection and parasitic diseases, and have slower growth rate and increased morbidity and mortality (Svoboda & Drabek, 2005).

## Conclusions

In conclusion, the results from these four experiments show that ferric citrate is a safe source of dietary Fe for broilers up to 2,000 mg/kg feed and up to 5,000 mg/kg for piglets. Additionally, ferric citrate is an easily digestible source of Fe in piglets and can be used alone or with other Fe sources.

## Supplemental Information

10.7717/peerj.12636/supp-1 Supplemental Information 1 Raw data from study A.

10.7717/peerj.12636/supp-2 Supplemental Information 2 Raw data from study B.

10.7717/peerj.12636/supp-3 Supplemental Information 3 Raw data from study C.

10.7717/peerj.12636/supp-4 Supplemental Information 4 Raw data from study D.

10.7717/peerj.12636/supp-5 Supplemental Information 5 Author checklist.

## Additional Information and Declarations

### Data Availability

The following information was supplied regarding data availability: The raw data analysed for all the parameters are available in the Supplemental Files.
